# Body mass index across adult life and cognitive function in the American elderly

**DOI:** 10.18632/aging.103209

**Published:** 2020-05-15

**Authors:** Yun Zhou, Tao Zhang, Daniel Lee, Lin Yang, Shengxu Li

**Affiliations:** 1 Department of Neurology, Beijing Tiantan Hospital, Capital Medical University, Beijing, China; 2 Department of Biostatistics, Shandong University School of Public Health, Jinan, China; 3 Children’s Minnesota Research Institute, Children’s Hospitals and Clinics of Minnesota, Minneapolis, MN 55404, USA; 4 Department of Cancer Epidemiology and Prevention Research, CancerControl Alberta, Alberta Health Services, Calgary, Canada; 5 Departments of Oncology and Community Health Sciences, Cumming School of Medicine, University of Calgary, Calgary, Canada

**Keywords:** cognitive function, body mass index, life course, NHANES

## Abstract

This study aimed to examine the associations of body mass index (BMI) across adult life with cognitive function in 2,637 participants aged 60 years or over from NHANES 2011-2014. The primary outcome was a composite score based on test scores on word list learning, animal naming, and digit symbol substitution. Exposures of interest included BMI at age 25, BMI 10 years before the survey, BMI at the survey (current BMI), and BMI burden calculated from age 25 to age at survey. BMI at age 25 was inversely associated with the composite score (β=-0.0271±0.0130 per kg/m^2^, P=0.038) and positively with low cognitive performance (odd ratio=1.04, 95% confidence interval: 1.01-1.07, P=0.010), defined as below 20 percentile of the composite score. Similar results were observed for BMI 10 years before the survey and BMI burden. Current BMI was positively associated with the composite score (β=0.0369±0.0113, P=0.001) and inversely associated with low cognitive performance (odd ratio=0.96, 95% confidence interval: 0.94-0.99, P=0.004). In conclusion, high BMI in early adult life is associated with low cognitive function in late life, which underscores the importance of a healthy body weight across the life course. The association between BMI and cognitive function at late life requires further investigation.

## INTRODUCTION

As world populations are rapidly aging, healthy aging has become a public health priority [1, 2]. An essential component of healthy aging is cognitive health [3]; conversely, cognitive impairments are a major threat to healthy aging. While older adults typically experience declines in cognitive functioning, adults over the age of 65 may experience cognitive impairments that compromise daily functions and undermine quality of life. Compromised cognitive function may contribute to or worsen other health conditions, and vice versa, starting a sustaining vicious cycle. Many factors, including but not limited to, age, race, education, adverse environments, unhealthy lifestyle (smoking and excessive drinking), early life exposure to cardiometabolic risk factors, including obesity, and chronic conditions, contribute to an accelerated decline in cognitive function in later life [2, 4, 5].

Obesity has a wide range of health consequences. However, evidence on the association between obesity and cognitive function in adults is inconsistent. Some studies support an inverse association between different obesity measures and cognitive function in young and middle-aged adults [6, 7], including individuals with normal weight obesity [8], while others support a positive association between BMI and cognitive function among older adults [9–12]. The Baltimore Longitudinal Study of Aging reported that obesity is associated with poorer performance in a variety of cognitive domains but enhanced performance on tests of attention and visuospatial ability [13].

The United States has experienced an obesity epidemic during the last four decades [14]. Hence, individuals living through the obesity epidemic since its onset are adults aged 60 years and older. Among these older adults, the influence of weight history across adult life with cognitive function late in life is not yet known. Such information is important for early prevention of late life cognitive impairment [15]. In this study, we aimed to examine the associations between body mass index (BMI) at different periods in adult life with cognitive function in late life among adults aged 60 or older from the National Health and Nutrition Examination Surveys (NHANES) 2011-2014.

## RESULTS

Table 1 describes the study sample by sex. Among 2,637 participants, 50.5% were female. Overall, 37.8% of the participants had obesity; of the BMI values at different times, current BMI was, on average, the highest among both men and women. Moreover, women tended to have higher cognitive function scores than men in CERAD Word Learning, CERAD Delayed Recall, and Digital Symbol Substitution, but not the Animal Fluency test.

**Table 1:** Characteristics of the study sample by sex: National Health and Nutritional Examination Survey (2011-2014).

**Variable**	**Male**	**Female**
n	1,307	1,330
Age group (%)		
60≤age<70 years	54.3	53.5
70≤age<80 years	29.5	30.0
≥80 years	16.2	16.5
Race		
Mexican American	8.9	7.2
Other Hispanic	9.1	9.2
Non-Hispanic White	47.3	51.9
Non-Hispanic Black	25.2	22.8
Other Race - Including Multi-Racial	9.5	8.9
BMI (kg/m^2^)		
At survey	28.6±5.6	29.4±6.9
1 year ago	28.1±5.6	28.3±7.0
10 years ago	27.5±5.1	27.4±6.3
At age 25 years	23.8±3.9	22.1±3.9
BMI burden from age 25 years to current	25.5±4.1	24.8±4.7
Obesity (%)	33.6	42.0
Average drinks per week	5.9±9.6	3.2±7.2
Education		
Less Than 9^th^ Grade	11.2	7.9
9-11^th^ Grade	13.2	13.5
High School Grad/GED or Equivalent	22.9	24.3
Some College or AA degree	26.2	32.7
College Graduate or above	26.5	21.6
Smoking (%)	15.1	10.6
Moderate recreational activity (%)	42.2	39.6
Vigorous recreational activity (%)	11.1	8.5
Asthma (%)	12.1	15.8
Congestive heart failure (%)	7.3	6.8
Stroke (%)	6.3	7.1
Cognitive function		
CERAD Word Learning	17.7±4.7	19.4±5.0
CERAD Delayed Recall	5.3±2.5	6.1±2.6
Animal Fluency Test	16.9±5.5	16.8±5.4
Digit Symbol Substitution Test	43.9±16.0	49.6±17.4

CERAD: the Consortium to Establish a Registry for Alzheimer’s Disease.

**Figure 1:**
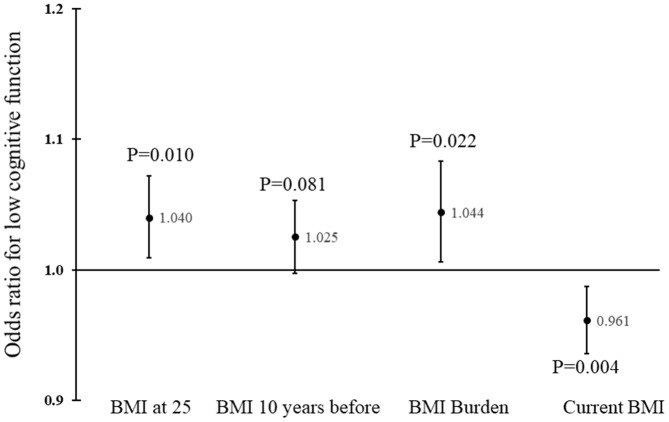
**Odds ratios and corresponding 95% confidence intervals for BMI at different times in adult life.** P values were adjusted for age, race, sex, education, physical activity, smoking, alcohol drinking, marital status, language used, and presence of major chronic diseases.

In the unadjusted model, BMI at age 25, BMI 10 years before the survey, and BMI burden were inversely associated with cognitive function, whereas current BMI was positively associated with cognitive function (Supplementary Table 2). In multiple regression analyses, BMI at age 25 (P=0.042), BMI 10 years before the survey (P=0.056), and BMI burden (P=0.046) were inversely associated with the composite score after adjusting for covariates and current BMI (Table 2). Similarly, they tended to be associated with low cognitive performance, with increase in BMI associated with increased odds of low cognitive performance (Figure 1).

**Table 2:** Associations of different cognitive functions with BMI at different times in adult life.

**Outcome**	**BMI at age 25**		**BMI 10 years ago**		**BMI burden**		**Current BMI**	
	**β±SE**	**P**	**β±SE**	**P**	**β±SE**	**P**	**β±SE**	**P**
Composite Score	-0.0271±0.0130	0.042	-0.0228±0.0120	0.056	-0.0320±0.0159	0.046	0.0369±0.0113	0.001
CERAD Word Learning	-0.0095±0.0047	0.059	-0.0071±0.0043	0.163	-0.0111±0.0057	0.089	0.0021±0.0029	0.058
CERAD Delayed Recall	-0.0071±0.0048	0.178	-0.0067±0.0044	0.180	-0.0088±0.0059	0.184	0.0101±0.0042	0.005
Animal Fluency Test	-0.0065±0.0047	0.200	-0.0054±0.0043	0.139	-0.0076±0.0057	0.151	0.0096±0.0029	0.002
Digit Symbol Substitution Test	-0.0057±0.0046	0.190	-0.0054±0.0040	0.197	-0.0071±0.0057	0.204	0.0127±0.0040	0.068

CERAD: the Consortium to Establish a Registry for Alzheimer’s Disease.

P values were adjusted for age, race, sex, education, alcohol drinking, smoking, moderate recreational activity, vigorous recreational activity, language used for the interview, asthma, congestive heart failure, and stroke.

Composite score was calculated as the sum of the individual scores that were standardized to z scores by inverse normal transformation.

Higher current BMI was associated with a higher value of the composite score of cognitive function (P=0.001), after adjusting for BMI burden and other covariates. Current BMI was also inversely associated with low cognitive performance, and each unit increase in current BMI was associated with 4% decrease in odds of low performance (Figure 1). Thus, lower BMI at age 25, 10 years before the survey, or lower cumulative burden, and higher current BMI was linked with higher cognitive functioning. Those who had BMI at age 25 below the median and current BMI above the median had the highest composite score; conversely, those who had BMI at age 25 above the median and current BMI below the median had the lowest composite score.

In sensitivity analyses, excluding those who were underweight or had extremely high BMI did not change the pattern of associations. Sex or age group differences were not observed.

## DISCUSSION

Our study demonstrates that current and historical accounts of BMI were linked with cognitive function in late life. That is, BMI at age 25 years, BMI 10 years before the survey, and cumulative burden across adult life were inversely associated with cognitive function in late life. Current BMI was positively associated with cognitive function, such that those who had higher BMI (above median) in early adult life and lower BMI (below median) in late life had the lowest level of cognitive function. Findings from the population-based sample reinforces the fact that exposure to obesity across the life course adversely influences cognitive function in late life.

As expected, we observed inverse associations of cognitive function with BMI at age 25 years, BMI 10 years before the survey, and cumulative burden. These observations are consistent with prior studies in young and midlife adults [8, 16, 19–21]. The mechanisms underlying the inverse associations between BMI across adult life and cognitive function in late life cannot be addressed by a cross-sectional study. There are, however, plausible mechanisms that may explain the observed associations. It is well established that obesity leads to significantly increased risk of cardiometabolic disorders, including insulin resistance/hyperglycemia, hypertension, dyslipidemia, sleep apnea, and inflammation [22–25]. These disorders, in turn, may accelerate cognitive declines and contribute to cognitive impairments in older adults [26–30]. Obesity over time also leads to increased risk of heart disease, stroke, liver disease, and type 2 diabetes, which can accelerate cognitive function decline [31–35].

The observed inverse associations of BMI in early adult life have important implications in preventing cognitive impairment in late life. As the global population is rapidly aging, medical and social burdens associated with declines in cognitive function will continue to rise. Preventing excessive body weight during young adulthood by promoting a healthy lifestyle, including sufficient physical activity, sleep, and a healthy diet, will mitigate the likelihood of cognitive functional impairment in late life. On the other hand, Hartanto et al. reported that the association between cognitive function and obesity is bidirectional, suggesting cognitive health-improving measures may prevent obesity and disrupt the vicious cycle between obesity and poor cognitive function [6]. It is known that BMI tracks over time from childhood to adult life [36] and that obesity in early life links to cognitive function in both early life and midlife [37, 38]. Therefore, prevention of obesity beginning in childhood should have long-term, multi-faceted benefits in reducing health burdens caused by cardiometabolic disorders and cognitive impairment in late life.

In contrast to the inverse associations between reported BMI at earlier ages and cognitive function, a positive association was observed between current BMI and cognitive function. The association was not confounded by poor cognitive function among underweight individuals because excluding underweight participants did not change the results. Similar observations of a positive association have been reported in older adults [9–12, 36, 37]. Of note, in a longitudinal study of 2,134 older adults (mean age = 77.9 years), lower baseline BMI was associated with faster cognitive decline six years later [10]. Even fat mass, assessed by dual-energy X-ray absorptiometry, showed similar associations with cognitive function [37]. It seems that the association between obesity and cognitive function changes across life course, as high BMI was protective for cognitive impairment only in adults 65 years or older, but not in adults 45-65 years in the same study [12]. It should be noted that not all studies reported positive associations in the elderly [38].

Several reasons may contribute to the apparently paradoxical associations with current BMI versus BMI during early adult life. Hormonal factors, such leptin and adiponectin, may contribute to the positive associations, as suggested by Gunathilake et al. [9]. Survival bias is also possible, but not likely, as associations were similar across the three age groups in the current study. Due to the cross-sectional nature of the study, understanding direct, causal effects between current BMI and cognitive function is not feasible. In the Health and Retirement Study, high baseline BMI in adults (average age of 58 years) was protective for cognitive impairment at follow-up [39]; however, low cognitive function predicted weight loss in the same study, suggesting a reverse causation [39]. It is also possible that low BMI reflects muscle loss in the elderly, which may contribute to the positive association observed in this study [40]. Similarly, low BMI might be an indication of frailty, a contributor to cognitive decline in the elderly [41, 42]. Additional research is needed to clarify the causal relationship between BMI and cognitive function in older adults.

The current study has several strengths and limitations. The study sample was a population-based sample with a relatively large sample size. NHANES data were collected with rigorous quality assurance/control protocols. Despite the cross-sectional study design, our study examined longitudinal associations between early life exposure to increased body weight and cognitive function in late life. We also used a composite score as the primary outcome, which covered multiple domains of cognitive function (Table 2). Nevertheless, we recognize that body weight during different periods in adult life was self-reported, introducing a potential for recall bias. It is important to mention, however, that recall bias, if existed, was more likely to be non-differential which would have biased the associations toward the null. Further, our exposure variable, BMI, does not differentiate fat mass from lean mass, and does not specify fat distribution. However, waist circumference, a measure of central obesity, produced similar results.

In conclusion, higher BMI in early and middle adult life is associated with poor cognitive function in later life. As obesity during adult and late adult life continues to rise, the inverse associations detected in our study underscores the importance of controlling body weight across the life course. The underlining reason for the paradoxical positive association between current BMI and cognitive function should be investigated in future studies with longitudinal data.

## MATERIALS AND METHODS

### Study samples

NHANES is a series of continuous surveys of national representative samples of the non-institutionalized general population of the United States, conducted by the National Center for Health Statistics (NCHS), to assess the health and nutritional status of adults and children (http://www.cdc.gov/nchs/nhanes.htm). Demographic, socioeconomic, dietary, and health-related data were collected by personal interviews. Information on weight history in adult life, including body weight at age 25 years, 10 years and one year before the interview, and height at age 25 years was obtained by the personal interview. Between 2011 and 2014, participants who were aged 60 years or older were eligible for cognitive function assessment. Those who had complete data on all cognitive function tests and on covariates (described later) were included in the study (n=2,637). The National Centers for Health Statistics Institutional Review Board reviewed and approved the study protocol (Protocol #2011-17) for the 2011-2014 surveys.

### Cognitive function tests

Details of the tests for cognitive function assessment have been described previously [4]. In brief, the CERAD Word Learning subtest (CERAD W-L) assesses immediate and delayed learning ability for new verbal information (memory sub-domain) and consists of three consecutive learning trials, and a delayed recall [16]. Immediately after reading aloud 10 unrelated words, participants tried to recall as many words as possible; the delayed word recall occurred after the other two cognitive exercises (to be described later) were completed (approximately 8-10 minutes from the start of the word learning trials). The number of word intrusions (incorrect words that were not on the list) for each word learning trial and the delayed word recall was also recorded. The Animal Fluency test examines categorical verbal fluency, a component of executive function [17]. Participants were asked to name as many animals as possible in one minute, with one point given for each named animal. The Digit Symbol Substitution test (DSST), a performance module from the Wechsler Adult Intelligence Scale (WAIS III), relies on processing speed, sustained attention, and working memory [18]. Participants were asked to copy in 2 minutes as many number-corresponding symbols as possible in 133 boxes on paper with keys at the top containing 9 numbers paired with symbols. The score corresponds to the total number of correct matches.

### Covariates

We included age, race, sex, education, alcohol use, smoking, physical activity, language used for the interview, and chronic conditions, which were all self-reported except for language used for the interview. Age was categorized in three groups: 60-69, 70–79, and 80 and over. Race/ethnicity was categorized into Mexican Americans, other Hispanic, Non-Hispanic White, non-Hispanic black, and other. Educational levels were categorized in less than 9^th^ grade, 9-11^th^ grade (including 12^th^ grade with no diploma), high school graduate/GED or equivalent, some college or an associate degree, and college graduate or above. Engaging moderate or vigorous recreational physical activity was separately recorded as a dichotomous variable. Moderate recreational activity was defined as any moderate-intensity sports, fitness, or recreational activities that caused a small increase in breathing or heart rate such as brisk walking, bicycling, swimming, or golf for at least 10 minutes continuously. Vigorous recreational activity was defined as any vigorous-intensity sports, fitness, or recreational activities that caused large increases in breathing or heart rate like running or basketball for at least 10 minutes continuously. The average number of alcohol drinks per week was used as a continuous variable. Smoking was coded as current vs. non-current smokers. Language used for the interview included English, Spanish, and other languages. We explored the following chronic conditions included in the medical conditions questionnaire as potential covariates: hypertension, diabetes, asthma, anemia, psoriasis, celiac disease, arthritis, gout, congestive heart failure, coronary heart disease, angina pectoris, heart attack, stroke, emphysema, thyroid problem, chronic bronchitis, liver condition, and cancer. Only asthma, heart failure, and stroke were included as covariates because only these three variables were associated with cognitive functions (P<0.05).

### Statistical analyses

The primary outcome variable was a composite cognitive function score derived from 4 individual scores according to the tests performed: CERAD word recall, CERAD delayed recall, the Animal Fluency test, and the DSST. The secondary outcomes included the scores from the individual tests. Scores for word recall and delayed recall were penalized by the number of word intrusions (the original score minus the number of word intrusions) before further manipulation. Each score of the individual tests was converted to a z-score by inverse-normal transformation. The composite cognitive function score was then calculated as the sum of the four z-scores. The exposure variables included BMI at age 25, BMI 10 years before the survey, and BMI burden across adult life. BMI burden was calculated as the total area of trapezoids, defined by BMIs at age 25, 10 years before the survey, 1 year before the survey, current BMI, and BMI at maximum and age at maximum when age at maximum was 25 or later, divided by the number of years (age at the survey minus 25). We did not include BMI one year before the survey as an exposure variable because it was closely correlated to current BMI (r=0.881; Supplementary Table 1). General linear models were used to examine the associations of outcomes with BMI at different times after adjusting for covariates. Logistic regression models were used to examine the associations of low cognitive function with exposures, adjusted for covariates; low cognitive function was defined as in the lowest 20^th^ percentile of the composite score. In models with BMI at age 25, BMI 10 years before the survey, or BMI burden as the exposure, current BMI was included in the model; in the model with current BMI was the exposure of interest, we adjusted for BMI burden. We also performed the following sensitivity analyses: 1) excluding those who were underweight (BMI<18.5 kg/m^2^) or had extremely high BMI (BMI>40kg/m^2^); 2) sex differences; and 3) age group differences (60-69, 70-79, and 80 or above). All analyses were performed with SAS Enterprise Guide 7.12 (SAS Institute Inc., Cary, NC).

## Supplementary Material

Supplementary Tables

## References

[1] Beard JR, Officer A, de Carvalho IA, Sadana R, Pot AM, Michel JP, Lloyd-Sherlock P, Epping-Jordan JE, Peeters GM, Mahanani WR, Thiyagarajan JA, Chatterji S. The World report on ageing and health: a policy framework for healthy ageing. Lancet. 2016; 387:2145–54. 10.1016/S0140-6736(15)00516-4 26520231 PMC4848186

[2] Toman J, Klímová B, Vališ M. Multidomain lifestyle intervention strategies for the delay of cognitive impairment in healthy aging. Nutrients. 2018; 10:1560. 10.3390/nu10101560 30347863 PMC6212852

[3] Anderson LA, McConnell SR. Cognitive health: an emerging public health issue. Alzheimers Dement. 2007 (2 Suppl); 3:S70–73. 10.1016/j.jalz.2007.01.018 19595979

[4] Brody DJ, Kramarow EA, Taylor CA, McGuire LC. Cognitive performance in adults aged 60 and over: National Health and Nutrition Examination Survey, 2011-2014. Natl Health Stat Report. 2019; 126:1–23. 31751207

[5] de Haan EH, Nys GM, Van Zandvoort MJ. Cognitive function following stroke and vascular cognitive impairment. Curr Opin Neurol. 2006; 19:559–64. 10.1097/01.wco.0000247612.21235.d9 17102694

[6] Hartanto A, Yong JC, Toh WX. Bidirectional associations between obesity and cognitive function in midlife adults: A Longitudinal Study. Nutrients. 2019; 11:2343. 10.3390/nu11102343 31581696 PMC6836311

[7] Wolf PA, Beiser A, Elias MF, Au R, Vasan RS, Seshadri S. Relation of obesity to cognitive function: importance of central obesity and synergistic influence of concomitant hypertension. The Framingham Heart Study. Curr Alzheimer Res. 2007; 4:111–16. 10.2174/156720507780362263 17430232

[8] Malandrino N, Capristo E, Taveira TH, Mingrone G, Wu WC. Cognitive function in individuals with normal weight obesity: Results from the Third National Health and Nutrition Examination Survey (NHANES III). J Alzheimers Dis. 2018; 65:125–35. 10.3233/JAD-180264 30010127

[9] Gunathilake R, Oldmeadow C, McEvoy M, Inder KJ, Schofield PW, Nair BR, Attia J. The association between obesity and cognitive function in older persons: how much is mediated by inflammation, fasting plasma glucose, and hypertriglyceridemia? J Gerontol A Biol Sci Med Sci. 2016; 71:1603–08. 10.1093/gerona/glw070 27075896

[10] Arvanitakis Z, Capuano AW, Bennett DA, Barnes LL. Body mass index and decline in cognitive function in plder black and white persons. J Gerontol A Biol Sci Med Sci. 2018; 73:198–203. 10.1093/gerona/glx152 28961897 PMC5861969

[11] Sturman MT, de Leon CF, Bienias JL, Morris MC, Wilson RS, Evans DA. Body mass index and cognitive decline in a biracial community population. Neurology. 2008; 70:360–67. 10.1212/01.wnl.0000285081.04409.bb 17881716

[12] Kim S, Kim Y, Park SM. Body mass index and decline of cognitive function. PLoS One. 2016; 11:e0148908. 10.1371/journal.pone.0148908 26867138 PMC4751283

[13] Gunstad J, Lhotsky A, Wendell CR, Ferrucci L, Zonderman AB. Longitudinal examination of obesity and cognitive function: results from the Baltimore longitudinal study of aging. Neuroepidemiology. 2010; 34:222–29. 10.1159/000297742 20299802 PMC2883839

[14] Flegal KM, Kruszon-Moran D, Carroll MD, Fryar CD, Ogden CL. Trends in obesity among adults in the United States, 2005 to 2014. JAMA. 2016; 315:2284–91. 10.1001/jama.2016.6458 27272580 PMC11197437

[15] Chen ST, Volle D, Jalil J, Wu P, Small GW. Health-promoting strategies for the aging brain. Am J Geriatr Psychiatry. 2019; 27:213–36. 10.1016/j.jagp.2018.12.016 30686664

[16] Morris JC, Heyman A, Mohs RC, Hughes JP, van Belle G, Fillenbaum G, Mellits ED, Clark C. The Consortium to Establish a Registry for Alzheimer’s Disease (CERAD). Part I. Clinical and neuropsychological assessment of Alzheimer’s disease. Neurology. 1989; 39:1159–65. 10.1212/WNL.39.9.1159 2771064

[17] Strauss E, Sherman EM, Spreen O. A Compendium of neuropsychological tests: Administration, norms and commentary. New York: Oxford University Press 2006.

[18] Salthouse TA. What do adult age differences in the Digit Symbol Substitution Test reflect? J Gerontol. 1992; 47:121–28. 10.1093/geronj/47.3.P121 1573192

[19] Wagner M, Grodstein F, Proust-Lima C, Samieri C. Long-term trajectories of body weight, diet, and physical activity from midlife through late-life and subsequent cognitive decline in women. Am J Epidemiol. 2019. [Epub ahead of print]. 10.1093/aje/kwz262 31781745 PMC7443200

[20] Masi S, Georgiopoulos G, Khan T, Johnson W, Wong A, Charakida M, Whincup P, Hughes AD, Richards M, Hardy R, Deanfield J. Patterns of adiposity, vascular phenotypes and cognitive function in the 1946 British Birth Cohort. BMC Med. 2018; 16:75. 10.1186/s12916-018-1059-x 29804545 PMC5971427

[21] Cook RL, O’Dwyer NJ, Donges CE, Parker HM, Cheng HL, Steinbeck KS, Cox EP, Franklin JL, Garg ML, Rooney KB, O’Connor HT. Relationship between obesity and cognitive function in young women: The Food, Mood and Mind Study. J Obes. 2017; 2017:5923862. 10.1155/2017/5923862 29291133 PMC5651104

[22] Després JP, Lemieux I, Bergeron J, Pibarot P, Mathieu P, Larose E, Rodés-Cabau J, Bertrand OF, Poirier P. Abdominal obesity and the metabolic syndrome: contribution to global cardiometabolic risk. Arterioscler Thromb Vasc Biol. 2008; 28:1039–49. 10.1161/ATVBAHA.107.159228 18356555

[23] Drager LF, Togeiro SM, Polotsky VY, Lorenzi-Filho G. Obstructive sleep apnea: a cardiometabolic risk in obesity and the metabolic syndrome. J Am Coll Cardiol. 2013; 62:569–76. 10.1016/j.jacc.2013.05.045 23770180 PMC4461232

[24] Klein S, Allison DB, Heymsfield SB, Kelley DE, Leibel RL, Nonas C, Kahn R, and Association for Weight Management and Obesity Prevention, and NAASO, and Obesity Society, and American Society for Nutrition, and American Diabetes Association. Waist circumference and cardiometabolic risk: a consensus statement from shaping America’s health: Association for Weight Management and Obesity Prevention; NAASO, the Obesity Society; the American Society for Nutrition; and the American Diabetes Association. Diabetes Care. 2007; 30:1647–52. 10.2337/dc07-9921 17360974

[25] Weihrauch-Blüher S, Schwarz P, Klusmann JH. Childhood obesity: increased risk for cardiometabolic disease and cancer in adulthood. Metabolism. 2019; 92:147–52. 10.1016/j.metabol.2018.12.001 30529454

[26] Hildreth KL, Grigsby J, Bryant LL, Wolfe P, Baxter J. Cognitive decline and cardiometabolic risk among Hispanic and non-Hispanic white adults in the San Luis Valley Health and Aging Study. J Behav Med. 2014; 37:332–42. 10.1007/s10865-013-9491-z 23329423

[27] Kesse-Guyot E, Julia C, Andreeva V, Fezeu L, Hercberg S, Galan P. Evidence of a cumulative effect of cardiometabolic disorders at midlife and subsequent cognitive function. Age Ageing. 2015; 44:648–54. 10.1093/ageing/afv053 25918184

[28] Kuo HK, Yen CJ, Chang CH, Kuo CK, Chen JH, Sorond F. Relation of C-reactive protein to stroke, cognitive disorders, and depression in the general population: systematic review and meta-analysis. Lancet Neurol. 2005; 4:371–80. 10.1016/S1474-4422(05)70099-5 15907742

[29] Lyall DM, Celis-Morales CA, Anderson J, Gill JM, Mackay DF, McIntosh AM, Smith DJ, Deary IJ, Sattar N, Pell JP. Associations between single and multiple cardiometabolic diseases and cognitive abilities in 474 129 UK Biobank participants. Eur Heart J. 2017; 38:577–83. 10.1093/eurheartj/ehw528 28363219 PMC5381595

[30] Papachristou E, Ramsay SE, Papacosta O, Lennon LT, Iliffe S, Whincup PH, Goya Wannamethee S. The Test Your Memory cognitive screening tool: sociodemographic and cardiometabolic risk correlates in a population-based study of older British men. Int J Geriatr Psychiatry. 2016; 31:666–75. 10.1002/gps.4377 26489874 PMC4855642

[31] Ampadu J, Morley JE. Heart failure and cognitive dysfunction. Int J Cardiol. 2015; 178:12–23. 10.1016/j.ijcard.2014.10.087 25464210

[32] Gottesman RF, Hillis AE. Predictors and assessment of cognitive dysfunction resulting from ischaemic stroke. Lancet Neurol. 2010; 9:895–905. 10.1016/S1474-4422(10)70164-2 20723846 PMC3592203

[33] Hajduk AM, Kiefe CI, Person SD, Gore JG, Saczynski JS. Cognitive change in heart failure: a systematic review. Circ Cardiovasc Qual Outcomes. 2013; 6:451–60. 10.1161/CIRCOUTCOMES.113.000121 23838109 PMC3872030

[34] Seo SW, Gottesman RF, Clark JM, Hernaez R, Chang Y, Kim C, Ha KH, Guallar E, Lazo M. Nonalcoholic fatty liver disease is associated with cognitive function in adults. Neurology. 2016; 86:1136–42. 10.1212/WNL.0000000000002498 26911638 PMC4820136

[35] Weinstein AA, de Avila L, Paik J, Golabi P, Escheik C, Gerber L, Younossi ZM. Cognitive performance in individuals with non-alcoholic fatty liver disease and/or type 2 diabetes mellitus. Psychosomatics. 2018; 59:567–74. 10.1016/j.psym.2018.06.001 30086995

[36] Kuo HK, Jones RN, Milberg WP, Tennstedt S, Talbot L, Morris JN, Lipsitz LA. Cognitive function in normal-weight, overweight, and obese older adults: an analysis of the Advanced Cognitive Training for Independent and Vital Elderly cohort. J Am Geriatr Soc. 2006; 54:97–103. 10.1111/j.1532-5415.2005.00522.x 16420204 PMC2834231

[37] Smith E, Bailey PE, Crawford J, Samaras K, Baune BT, Campbell L, Kochan N, Menant J, Sturnieks DL, Brodaty H, Sachdev P, Trollor JN. Adiposity estimated using dual energy X-ray absorptiometry and body mass index and its association with cognition in elderly adults. J Am Geriatr Soc. 2014; 62:2311–18. 10.1111/jgs.13157 25516027

[38] Han C, Jo SA, Seo JA, Kim BG, Kim NH, Jo I, Park MH, Park KW. Adiposity parameters and cognitive function in the elderly: application of “Jolly Fat” hypothesis to cognition. Arch Gerontol Geriatr. 2009; 49:e133–38. 10.1016/j.archger.2008.11.005 19108905

[39] Suemoto CK, Gilsanz P, Mayeda ER, Glymour MM. Body mass index and cognitive function: the potential for reverse causation. Int J Obes. 2015; 39:1383–89. 10.1038/ijo.2015.83 25953125 PMC4758694

[40] van Dam R, Van Ancum JM, Verlaan S, Scheerman K, Meskers CG, Maier AB. Lower cognitive function in older patients with lower muscle strength and musclem mass. Dement Geriatr Cogn Disord. 2018; 45:243–50. 10.1159/000486711 29913450 PMC6067649

[41] Nishiguchi S, Yamada M, Fukutani N, Adachi D, Tashiro Y, Hotta T, Morino S, Shirooka H, Nozaki Y, Hirata H, Yamaguchi M, Arai H, Tsuboyama T, Aoyama T. Differential association of frailty with cognitive decline and sarcopenia in community-dwelling older adults. J Am Med Dir Assoc. 2015; 16:120–24. 10.1016/j.jamda.2014.07.010 25244957

[42] Ma L, Zhang L, Sun F, Li Y, Tang Z. Cognitive function in prefrail and frail community-dwelling older adults in China. BMC Geriatr. 2019; 19:53. 10.1186/s12877-019-1056-8 30813907 PMC6391822

