# Supplementary material for: Body mass index across adult life and cognitive function in the American elderly
Source: Aging (Albany NY). 2020 May 15;12(10):9344–53. doi: 10.18632/aging.103209 (PMC7288936; doi:10.18632/aging.103209)
Supplement: Supplementary Tables [file aging-12-103209-s001..pdf]

SUPPLEMENTARY TABLES

Supplementary Table 1. Correlations of BMI across different time points.

	BMI at 25	BMI 10 years before survey	BMI 1 year before survey	BMI burden
BMI 10 years before survey	0.560			
BMI 1 year before survey	0.492	0.796		
BMI burden	0.779	0.950	0.828	
Current BMI	0.395	0.727	0.881	0.732

P<0.00001 for all correlation coefficients.

Supplementary Table 2. Correlations of BMI across different time points with cognitive function measures.

Outcome	BMI at age 25		BMI 10 years before		BMI burden		Current BMI	
	Correlation coefficient	P	Correlation coefficient	P	Correlation coefficient	P	Correlation coefficient	P
Composite Score	-0.049	0.011	-0.025	0.199	-0.029	0.143	0.046	0.018
CERAD Word Learning	-0.057	0.003	-0.018	0.350	-0.028	0.150	0.036	0.068
CERAD Delayed Recall	-0.037	0.054	-0.001	0.970	-0.006	0.750	0.054	0.005
Animal Fluency Test	0.002	0.923	-0.003	0.892	0.005	0.815	0.050	0.010
Digit Symbol Substitution Test	-0.062	0.002	-0.057	0.004	-0.060	0.002	0.004	0.824
